# Technical note: Atlas‐based Auto‐segmentation of masticatory muscles for head and neck cancer radiotherapy

**DOI:** 10.1002/acm2.13008

**Published:** 2020-08-25

**Authors:** Xiangguo Zhang, Haihui Chen, Wen Chen, Brandon A. Dyer, Quan Chen, Stanley H. Benedict, Shyam Rao, Yi Rong

**Affiliations:** ^1^ Department of Radiation Oncology University of California Davis Medical Center Sacramento CA USA; ^2^ Department of Radiation Oncology The Affiliated Yuebei People’s Hospital of Shantou University Medical College Shaoguan China; ^3^ Department of Radiation Oncology Liuzhou Worker's Hospital Liuzhou Guangxi China; ^4^ Department of Radiation Oncology Xiangya Hospital of Central South University Changsha China; ^5^ Department of Radiation Oncology University of Washington Seattle WA USA; ^6^ Department of Radiation Oncology University of Kentucky Lexington KY USA

**Keywords:** atlas‐based auto‐segmentation, head and neck cancer, masticatory muscles, radiation therapy

## Abstract

**Purpose:**

The study aimed to use quantitative geometric and dosimetric metrics to assess the accuracy of atlas‐based auto‐segmentation of masticatory muscles (MMs) compared to manual drawn contours for head and neck cancer (HNC) radiotherapy (RT).

**Materials and methods:**

Fifty‐eight patients with HNC treated with RT were analyzed. Paired MMs (masseter, temporalis, and medial and lateral pterygoids) were manually delineated on planning computed tomography (CT) images for all patients. Twenty‐nine patients were used to generate the MM atlas. Using this atlas, automatic segmentation of the MMs was performed for the remaining 29 patients without manual correction. Auto‐segmentation accuracy for MMs was compared using dice similarity coefficients (DSCs), Hausdorff distance (HD), HD95, and variation in the center of mass (∆COM). The dosimetric impact on MMs was calculated (∆dose) using dosimetric parameters (D99%, D95%, D50%, and D1%), and compared with the geometric indices to test correlation.

**Results:**

DSC_mean_ ranges from 0.79 ± 0.04 to 0.85 ± 0.04, HD_mean_ from 0.43 ± 0.08 to 0.82 ± 0.26 cm, HD95_mean_ from 0.32 ± 0.08 to 0.42 ± 0.16 cm, and ∆COM_mean_ from 0.18 ± 0.11 to 0.33 ± 0.23 cm. The mean MM volume difference was < 15%. The correlation coefficient (r) of geometric and dosimetric indices for the four MMs ranges between −0.456 and 0.300.

**Conclusions:**

Atlas‐based auto‐segmentation for masticatory muscles provides geometrically accurate contours compared to manual drawn contours. Dose obtained from those auto‐segmented contours is comparable to that from manual drawn contours. Atlas‐based auto‐segmentation strategy for MM in HN radiotherapy is readily availalbe for clinical implementation.

## 1 Introduction

Radiation therapy (RT) plays an essential role in the management of patients with head and neck cancer (HNC).^1^ With improved survival outcomes in the setting of pathogen‐associated HNCs, reducing long‐term toxicity is increasingly important. Specifically, trismus due to RT‐induced masticatory muscles (MMs) injury is a common clinical complication for patients with HNC treated with RT and has a significant impact on health‐related quality of life.^2^, ^3^, ^4^ However, contouring of these muscles as dose‐limiting structures during radiotherapy treatment planning is not routine. Due to low soft‐tissue contrast and lack of clear boundaries on typical planning CT images, manual segmentation of these muscles as organs‐at‐risk (OARs) is challenging. Thus, manual segmentation for these muscles is subject to large interuser variability and is time consuming.^5^, ^6^ As a result, RT dose tolerance levels to MMs have not been well studied. To reduce toxicity and improve long‐term patient swallowing outcomes and quality of life, dose constraints for the MMs need to be established and accurate, consistent delineation of MMs is necessary.

In recent years, with improved technology for medical image analysis, computer‐aided fully or semiautomatic segmentation techniques have shown promise in radiation oncology to provide fast and accurate OAR segmentation.^7^, ^8^, ^9^ Atlas‐based auto‐segmentation is an important, automatic segmentation technique which uses atlas templates built from previously validated OAR contours to automatically create contours for new patients.^9^, ^10^, ^11^ The key component of auto‐segmentation is a database (i.e., the so‐called atlas) containing image data with OAR segmentations. The atlas contours are then propagated to the image data of a new patient via rigid and deformable image registrations. Several studies have evaluated the use of atlas‐based auto‐segmentation to reduce contouring time and inter‐ and intraobserver variations in OARs contouring for HNC.^5^, ^12^ A few studies^13^, ^14^, ^15^ evaluated atlas‐based algorithms in delineating masticatory muscles for HNC patients. However, these studies evaluated their in‐house algorithms, which cannot be directly translated to clinical practice and are not widely available. Furthermore, the dataset sample sizes for the previous studies are relatively low. Teguh et al.^13^ used ten cases for building the atlas and 12 for testing. Additionally, the dosimetric impact of using auto‐segmented contours has not been thoroughly investigated.

We assessed the feasibility of using a commercial atlas‐based algorithm for segmenting MMs for HNC patients in a large patient cohort. We also validated the geometric and dosimetric accuracy of auto‐segmented contours against manual segmentations performed by experienced HNC radiation oncologists. The major significance of the present study is to establish efficient and accurate MM auto‐segmentation strategy in the clinical workflow for improving dose–volume assessment. The proposed approach and results are widely available and deployable, thus enabling for future evaluation and optimization of treated patients’ Quality of Life (QoL).

## 2 Methods and Materials

### 2.A Patients

This single‐center retrospective study was performed following Institutional Research Board (IRB) committee approval. A total of 58 patients who received RT treatment with pathologically confirmed squamous cell HNC without MM invasion were included. Demographic data are shown in Table 1. There were 27 patients aged more than 60 yr, and 44 patients were men. Disease sites include 37 cases of oropharynx cancer, seven of larynx cancer, six of nasopharynx and sinonasal cancer, and eight of cancers of other sites. Twenty‐four patients had T3/T4 disease; 37 patients had N + disease. All patients were staged I–IV according to the 8th AJCC staging system. The prescription dose for high‐risk regions ranges from 60 Gy to 70 Gy, which was delivered via volumetric modulated arc therapy (VMAT).

**Table 1:** Demographic characteristics of patients.

Characteristic	Patients for validation n（%）	Patients for building atlas n（%）
Gender		
Male	21 (72.4)	23 (79.3)
Female	8 (27.6)	6 (20.7)
Age		
≤60 years	15 (51.7)	16 (55.2)
＞60 years	14 (48.3)	13 (44.8)
Primary site		
Oropharynx	20 (68.97)	17 (58.62)
Larynx	4 (13.79)	3 (10.34)
Nasopharynx and sinonasal	2 (6.90)	4 (13.79)
Other cancers	3 (10.34)	5 (17.24)
T stage (AJCC 08th)		
1	5 (17.24)	6 (20.69)
2	9 (31.03)	9 (31.03)
3	7 (24.14)	4 (13.79)
4	6 (20.69)	7 (24.14)
N/X	2 (6.90)	3 (10.34)
N stage		
0	7 (24.14)	13 (44.83)
1	8 (27.59)	4 (13.79)
2	13 (44.83)	10 (34.48)
3	0 (0.00)	2 (6.90)
N/X	1 (3.45)	0 (0.00)
Overall stage		
Ⅰ	2 (6.90)	4 (13.79)
Ⅱ	3 (10.34)	4 (13.79)
Ⅲ	6 (20.69)	5 (17.24)
Ⅳ	17 (58.62)	16 (55.17)
N/X	1 (3.45)	0 (0.00)

### 2.B Target delineation

All patients were immobilized in the supine position using a head, neck, and shoulder thermoplastic mask with MoldCare pillow and head holder. Simulation CT images with contrast were obtained prior to RT on a Philips Brilliance Big Bore scanner (Amsterdam, Netherlands). Standardized CT scan covers a region from the head to 2 cm below sternoclavicular joint. The transverse field of view was 512*512; pixel size was 1.183 mm * 1.183 mm with a thickness of 3.0 mm. All images were de‐identified and imported into Raystation treatment planning system version 6.0 (RaySearch Laboratory AB, Stockholm, Sweden). Manual segmentation of the four paired MMs (masseter, temporalis, and medial and lateral pterygoids) was completed by a radiation oncology attending specialized in HNC according to the institutional guidelines and published recommendations.^16^ Segmentations were reviewed, edited when needed, and confirmed by a senior HNC radiation oncology attending.

### 2.C Atlas‐based algorithms

Patient data were divided into two groups for atlas construction (n = 29) and atlas validation (n = 29). A multi‐atlas‐based autosegmented algorithm (MABS) in Raystation was used to generate auto‐segmented contours, and multiple atlas templates were fused for testing image datasets.^17^ Raystation deformable image registration (DIR) algorithms were used to fuse and deform the two images and propagates contours from multiple datasets to the testing image for AS. Raystation's ANAtomically Constrained Deformation Algorithm (ANACONDA) was used for DIR. ANACONDA combines image intensity information with anatomical information in calculating deformation vectors to achieve best match between images.^18^

### 2.D Volume comparison and overlap analysis

Four pairs of MMs (eight total muscles) were evaluated, including masseter‐right (M‐R), masseter‐left (M‐L), temporalis‐right (T‐R), temporalis‐left (T‐L), lateral pterygoid‐right (LP‐R), lateral pterygoid‐left (LP‐L), medial pterygoid‐right (MP‐R), and medial pterygoid‐left (MP‐L). Geometric indices for manual and auto‐segmented contours were calculated for each MM, including absolute volume difference, dice similarity coefficient (DSC), Hausdorff distance (HD), HD95, and center of mass (∆COM) displacement^19^. Quantitative volume comparison was defined as:

$$\Delta V_{\mathrm{mean}}=\left|\frac{\left(V_{mean,MS}‐V_{mean,AS}\right)}{V_{mean,MS}}\right|$$

where Vmean,_MS_ defines the mean manual segmentation volume, and Vmean,_AS_ defines to the mean auto‐segmentation volume.

Dice similarity coefficient (DSC) is a geometric volumetric similarity measure used to determine the degree of overlap of two set of contours, which provides a value that simultaneously quantifies differences in volume size and orientation for nonsymmetric shape of contours. DSC normalizes the intersection volume to a value between 0 (no overlap) and 1 (perfect overlap) and is defined as:

$$DSC=\frac{2\left|\text{Vm}\cap \text{Va}\right|}{\left|\text{Vm}\right|+\left|\text{Va}\right|}$$

where Vm and Va are the volumes of the manual drawn and auto‐segmented contours, respectively^20^.

The Hausdorff distance is another measure of relative contour overlap and defines as the maximum distance of the same object.

$\text{Hausdorff distance}\;\left(\text{HD}\right)=\max\left\{\begin{array}{c} \text{min d}\left(a\right)\\ a\in A \end{array},\begin{array}{c} \text{min d}\left(b\right)\\ b\in B \end{array}\right\}$“a” and “b” represent the points on contour A and contour B. where $\min_{a\in A}d\left(a\right)$ is the minimum distance of all points on the contour A to points on the contour B, so as the same definition used for $\min_{b\in B}d\left(b\right)$. The HD95 is the 95th percentile distance over all distances from points in A to their closest point in B. It was used to minimize the impact of large outliers in the HD calculation on the overall data. While the center of mass displacement(∆COM) is to evaluate the overall shift between two contours. It is defined as:

$$\Delta \text{COM}\;=\sqrt[{2}]{{\left(x1‐x2\right)}^{2}+{\left(y1‐y2\right)}^{2}+{\left(z1‐z2\right)}^{2}}$$

x(1,2), y(1, 2), and z(1,2) were indicate the coordinates of the selected reference contours, which is same as the geometric centroid of the contour.

### 2.E Dosimetric comparison

Dose–volume information for MM structures was compared, including D99% (the minimum absorbed dose, cGy), D95% (the prescribed dose, cGy), D50% (the median absorbed dose, cGy), and D1% (the maximum absorbed dose, cGy). Dose difference between manual and auto‐segmented contours for MMs was calculated as:

$$\Delta dose=\left|\frac{\left(dose_{\mathrm{MS}}‐dose_{\mathrm{AS}}\right)}{dose_{\mathrm{MS}}}\right|$$

with dose_MS_ defines the dose of manual segmentation contours, and dose_AS_ defines the dose of auto‐segmentation contours.

### 2.F Correlation of volume accuracy with plan quality

To explore the correlation between contour accuracy and plan quality, geometric indices were analyzed with respect to dosimetric endpoints for every MM. The correlation between geometric indices and dose difference was evaluated using Pearson's correlation coefficient (r).

## 3 Results

**Fig. 1:**
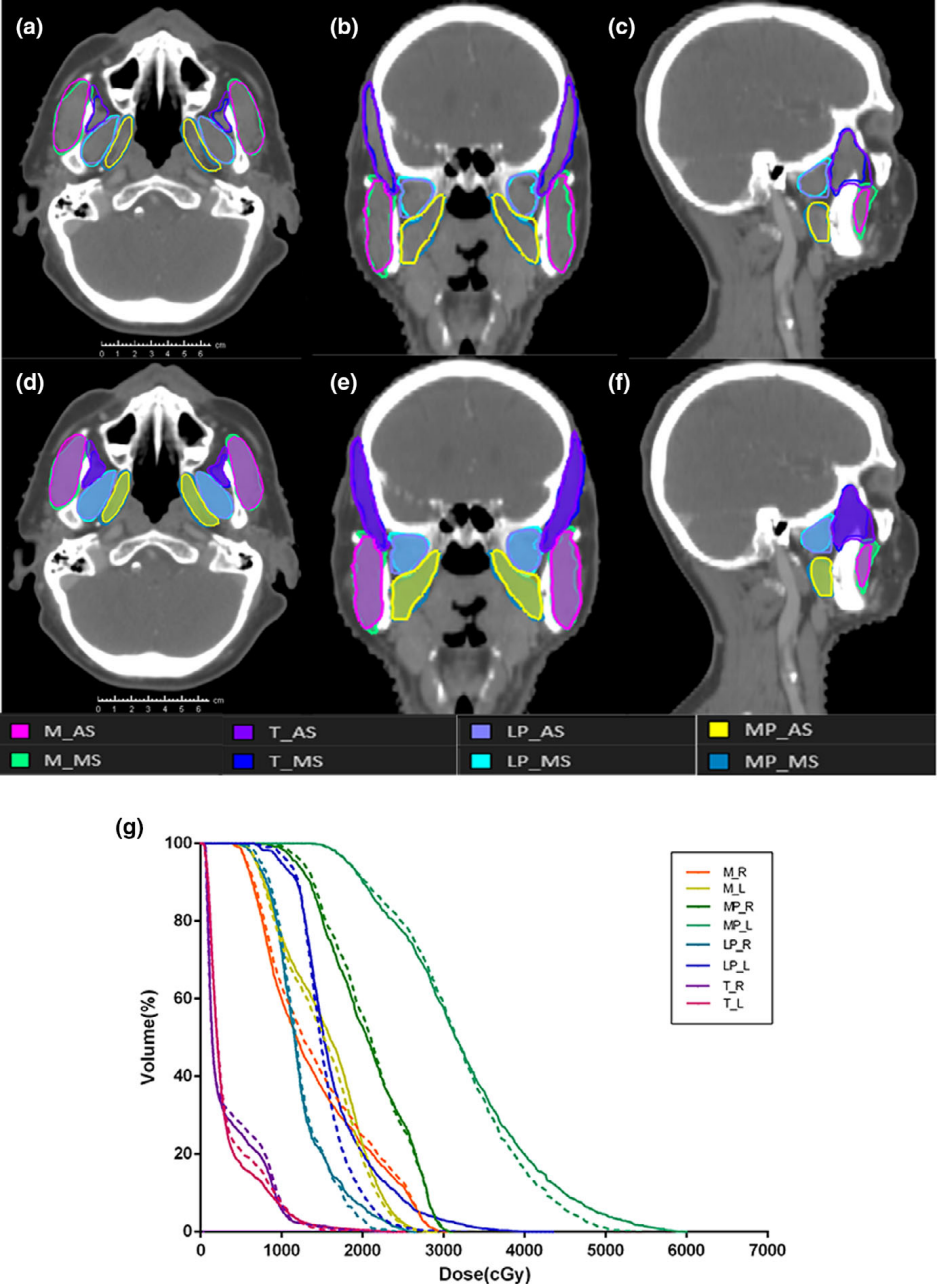
(a) Representative CT images of manual drawn and auto‐segmented contours for eight mastication muscles, respectively. A–C show manual contours (solid lines) and D–F show auto‐segmented contours (color wash). A and D: transverse section; B and E: coronal section; C and F: sagittal section. Masseter (M), temporalis (T), lateral pterygoid (LP), medial pterygoid (MP); (b) dose–volume histogram of eight mastication muscles for manual drawn (solid lines) and auto‐segmented (dashed lines) contours for the patient in Fig. 1. Masseter‐right (M‐R), masseter‐left (M‐L), temporalis‐right (T‐R), temporalis‐left (T‐L), lateral pterygoid‐right (LP‐R), lateral pterygoid‐left (LP‐L), medial pterygoid‐right (MP‐R), and medial pterygoid‐left (MP‐L).

**Fig. 2:**
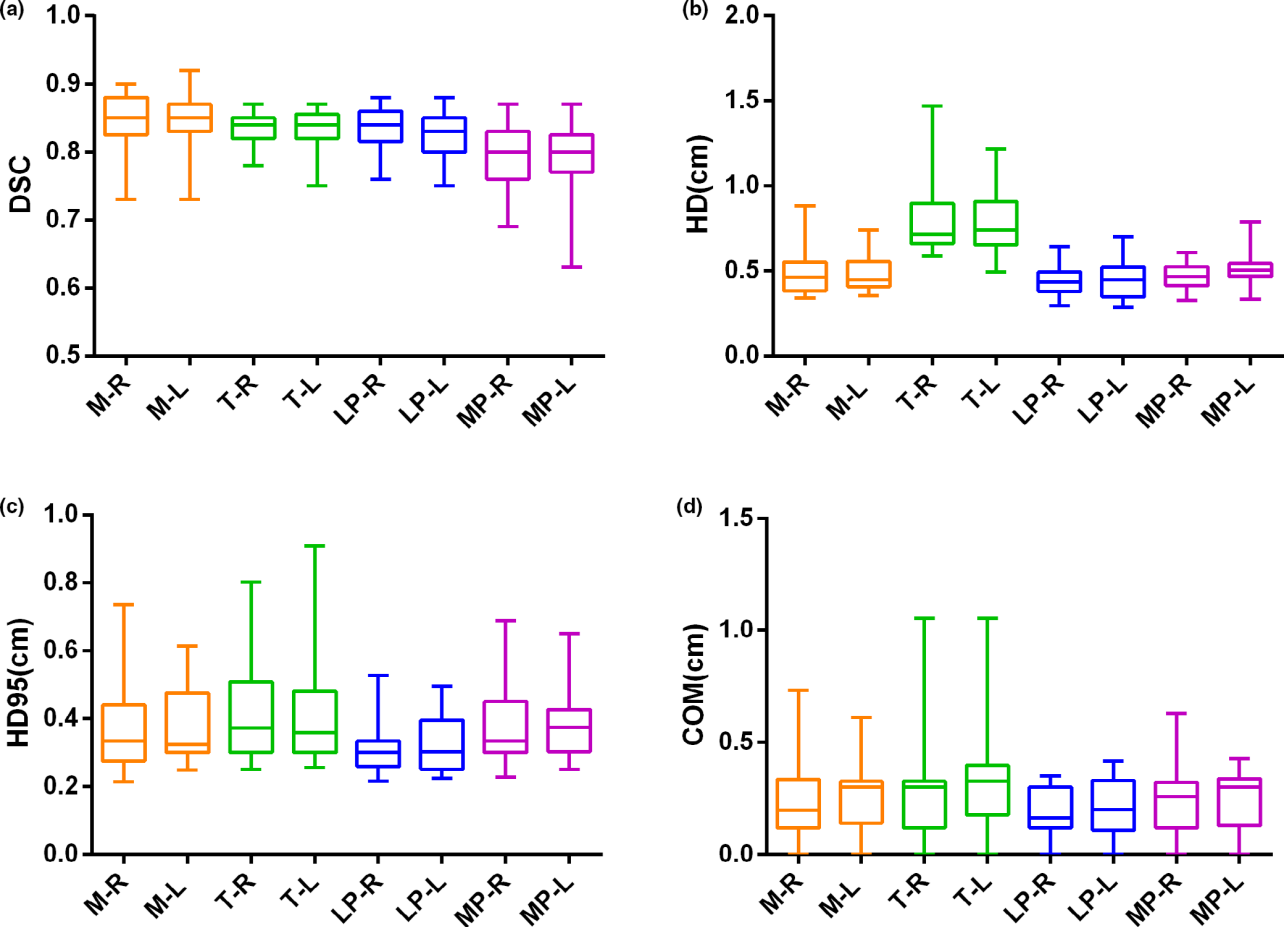
Box plots show comparison of DSC (a), HD (b), HD95 (c), and △COM (d) geometric parameters for the four pairs of MMs from 29 validation patients. The limits of each box represent the 25th and 75th percentiles, the middle black line represents the median, and the upper and lower whiskers represent the highest and lowest values, respectively.

A representative example of manual and auto‐segmented contours is shown in Fig. 1(a). Visually, there is an excellent agreement for each MM. Figure 1(b) shows DVHs for the patient in Fig. 1(a). For the 29 test patients, the geometric comparison indices are shown in Fig. 2 and Table 2. Figure 2 shows DSC, HD, HD95, △COM values for masticatory muscles with manual and auto‐segmented structures in the validation cohort. The box plot shows that the masseter had the highest DSC value, while the medial pterygoid MP achieved the lowest DSC value. For HD/HD95, the temporalis had the highest value, while the lateral pterygoid had the lowest value. The minimum △COM value was observed with the lateral pterygoid, and the maximum △COM was observed with the temporalis.

**Table 2:** Geometric indices comparing manual drawn and auto‐segmented contours.

	M‐R	M‐L	T‐R	T‐L	LP‐R	LP‐L	MP‐R	MP‐L
DSC	0.85 ± 0.04	0.84 ± 0.04	0.84 ± 0.02	0.83 ± 0.03	0.83 ± 0.03	0.82 ± 0.03	0.79 ± 0.04	0.79 ± 0.05
HD (cm)	0.49 ± 0.12	0.48 ± 0.10	0.82 ± 0.26	0.78 ± 0.19	0.43 ± 0.08	0.44 ± 0.11	0.57 ± 0.54	0.51 ± 0.09
COM (cm)	0.24 ± 0.16	0.27 ± 0.14	0.30 ± 0.23	0.33 ± 0.23	0.18 ± 0.11	0.22 ± 0.15	0.23 ± 0.14	0.25 ± 0.13
X axis	0.16 ± 0.17	0.12 ± 0.20	0.20 ± 0.22	0.23 ± 0.22	0.09 ± 0.14	0.12 ± 0.15	0.15 ± 0.17	0.17 ± 0.15
Y axis	0.11 ± 0.09	0.16 ± 0.17	0.14 ± 0.16	0.15 ± 0.15	0.08 ± 0.07	0.09 ± 0.10	0.07 ± 0.07	0.10 ± 0.07
Z axis	0.06 ± 0.08	0.12 ± 0.110	0.06 ± 0.10	0.10 ± 0.11	0.05 ± 0.07	0.07 ± 0.07	0.09 ± 0.08	0.06 ± 0.09
△V (cm^3^)	0.14 ± 0.14	0.07 ± 0.08	0.12 ± 0.07	0.10 ± 0.06	0.09 ± 0.08	0.12 ± 0.06	0.12 ± 0.07	0.13 ± 0.08

Table 2 shows the DSC, HD, HD95%, and △COM comparing manual versus autosegmented contours. DSC results show a high degree of geometric overlap, with DSC ranging from 0.79 ± 0.05 to 0.85 ± 0.04. The largest HD values were observed for the temporalis, where T‐R = 0.82 (±0.26) cm and T‐L = 0.78 (±0.19) cm. The lateral pterygoid achieved the lowest HD value, where LP‐R = 0.43 (±0.08) cm and LP‐L = 0.44 (±0.11) cm. The HD values in cm for M‐R, M‐L, MP‐R, and MP‐L are 0.49 (±0.12), 0.48 (±0.10), 0.57 (±0.54), and 0.51 (±0.09), respectively. Similarly, the temporalis has the highest HD95 value (cm), while the lateral pterygoid has the lowest HD95 value. The overall ∆COM_mean_ range was 0.18–0.33 cm, with 0.24 (±0.16) cm for M‐R, 0.27 (±0.14) cm for M‐L, 0.30 (±0.23) cm for T‐R, 0.33 (±0.23) cm for T‐L, 0.18 (±0.11) cm for LP‐R, 0.22 (±0.15) cm for LP‐L, 0.23 (±0.14) cm for MP‐R, and 0.25 (±0.13) cm for MP‐L. The mean difference in volume was < 15%. The highest difference was observed for the M‐R volumes with ΔV of 14% (±14%), while M‐L had the lowest difference with ΔV = 7% (±8%).

**Fig. 3:**
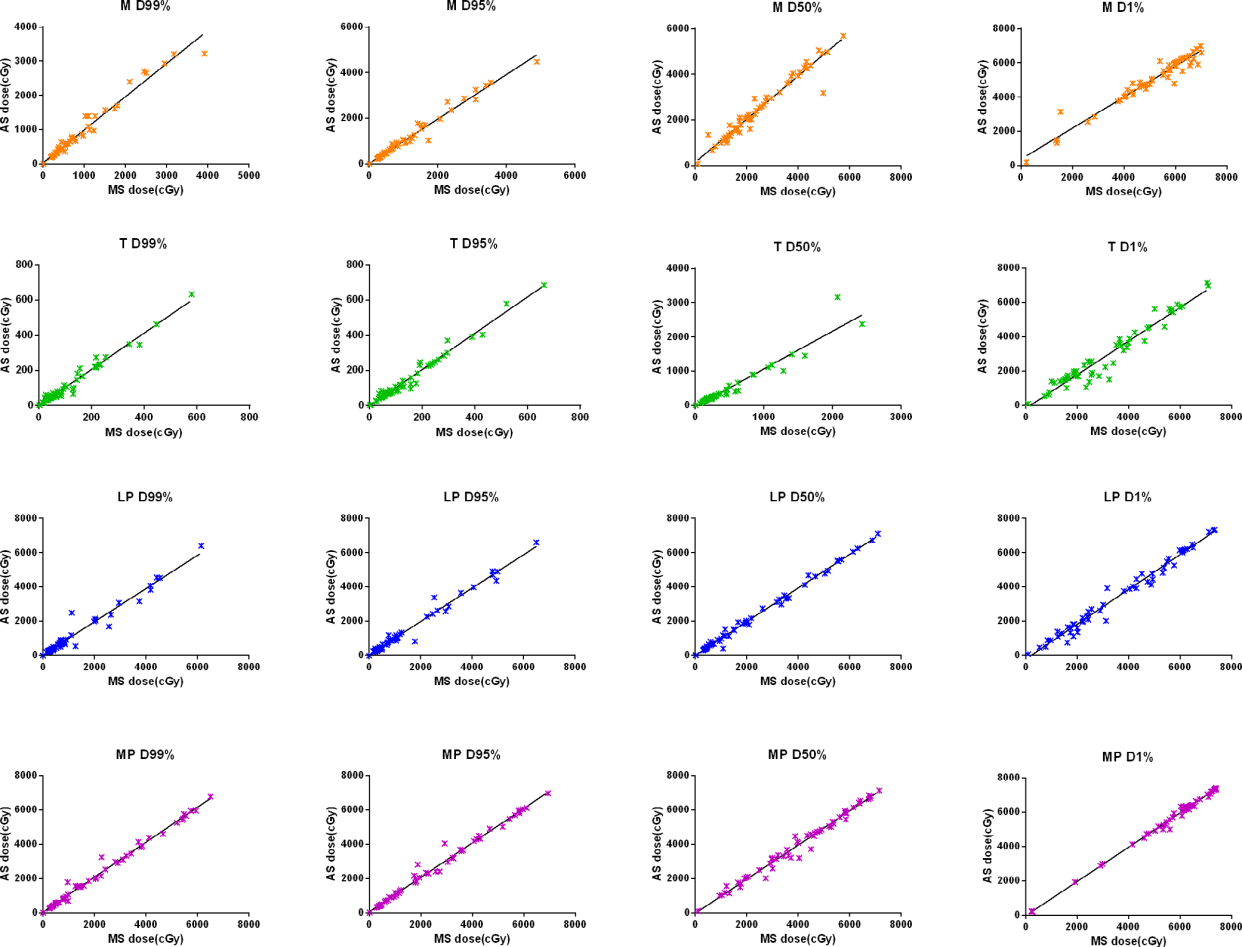
Comparison of dose parameters for manual and auto‐segmented contours. Plots show the D99%, D95%, D50%, and D1% of masseter (M), temporalis (T), lateral pterygoid (LP), and medial pterygoid (MP) for all patients. Solid line represents the linear regression.

Table 3 shows that the observed correlation coefficient of geometric and dosimetric indices for MMs. No strong correlation was found amongst geometric indices, while week correlation was observed between some geometric indices and dose differences. The absolute percentage dose differences between manual and auto‐segmented contours (difference normalized to the dose value of manual contours) for each MM pair are tabulated in Table 4. The dose–volume parameters evaluated include D99, D95, D50, and D1. The percentage dose differences for all four dose volume parameters are mostly less than 15% for all MM contours, with higher average dose deviations observed with temporalis and lateral pterygoid muscles. To further elaborate the differences, Fig. 3 shows a linear regression between manual and auto‐segmented contours for the four pairs of muscles, with the above‐mentioned four dose–volume indices in comparison. Dose variation is observed between manual and auto‐segmented contours, especially for muscles ipsilateral and in proximity to tumor.

**Table 3:** Pearson's correlation r for geometric measures and normalized dose differences between manual and auto segmented contours.

	DSC	HD	COM	**△**V
DSC	NA	−0.4341	−0.4558	−0.0676
HD	−0.4341	NA	0.3003	0.1334
COM	−0.4558	0.3003	NA	−0.1217
△V	−0.0676	0.1334	−0.1217	NA
**△**D99%	0.1910	−0.0257	0.0378	0.1131
**△**D95%	−0.0697	−0.0655	0.2233	0.0681
**△**D50%	0.0933	0.0085	−0.0791	−0.0020
**△**D1%	0.0091	0.0025	−0.1448	0.1357

Dose parameters denote the absolute value of the difference between manual and auto‐segmented contours normalized to the dose values from the manual contours.

**Table 4:** The percent dose difference (Mean ± SD) between manual and auto‐segmented contours.

∆dose (%)	M‐R	M‐L	T‐R	T‐L	LP‐R	LP‐L	MP‐R	MP‐L
∆D99	8.5 ± 7.8	8.4 ± 11.2	13.6 ± 17.9	15.8 ± 24.8	11.5 ± 13.0	16.7 ± 23.6	4.7 ± 6.6	8.8 ± 16.7
∆D95	6.3 ± 7.4	7.4 ± 7.9	11.5 ± 15.7	12.6 ± 17.7	10.5 ± 14.5	12.7 ± 15.4	3.7 ± 4.6	7.8 ± 11.1
∆D50	5.4 ± 5.9	12.8 ± 30.9	6.8 ± 8.0	9.2 ± 11.1	6.4 ± 9.6	9.0 ± 14.0	4.9 ± 6.3	5.0 ± 65
∆D1	4.1 ± 4.0	6.7 ± 19.0	11.0 ± 13.4	13.3 ± 14.9	8.3 ± 10.0	9.6 ± 11.9	1.2 ± 1.5	2.0 ± 44

## 4 Discussion

In this study, we assessed the performance of atlas‐based segmentation for masticatory muscles compared with manual segmentation. Quantitative assessment included geometric and dosimetric accuracy of manual versus automated segmentation. We found that atlas‐based auto‐segmentation of masticatory muscles results in high geometric overlap with manual contours and low, nonsignificant dosimetric differences. The validated atlas‐based AS algorithm can be used toward improving clinical workflow efficiency and help with delineating normal structures where manual segmentation is not feasible due to large user variations or time constraints. Furthermore, a commercially available registration and deformation platform was used in this study; thus, the created atlas could be widely employed and freely available for users with the same platform.

Prior studies have investigated the use of in‐house atlas‐based auto‐segmentation techniques for MMs delineation. Teguh et al.^13^ used ten HNC patients for building atlas, and 12 for testing. They showed that DSC between auto‐contours and manual contours had a mean of 0.71 for masticatory muscles using a multiple‐subject approach. Han et al.^14^ tested ten HNC patients by using two atlas selection strategies, and found that the median DSC is below 0.8 for the masseter muscles and pterygoid muscles using a single atlas, but over 0.8 using a multi‐atlas strategy. Hague et al.^15^ studied multiple‐subject auto‐segmentation of MMs for five patients found that the similarity of the atlas‐based autosegmented contours with their reference outlines was satisfactory with a mean DSC of 0.8 (±0.1) for LP, 0.7 (±0.2) for MP, 0.8 (±0.1) for T, and 0.9 (±0.1) for M. In general, our DSC results are equivalent or superior to these prior studies, mostly due to our multi‐atlas‐based segmentation algorithm approach, as well as the large patient testing cohort. To further evaluate the performance of atlas segmentation, ΔCOM, the mean delineated volumes change, and HD/HD95 were assessed and compared to the manual drawn reference for each MM volume. All metrics showed that the auto‐segmented contours agree well with the manual contours. Consistent with our current results, the study by Hague et al.^15^ also found that auto‐segmentation technique can achieve low ΔCOM values for temporalis with 0.45 ± 0.30 cm for X axis, 0.34 ± 0.28 cm for Y axis, and 0.21 ± 0.14 cm for Z axis.

Previous studies have shown^21^, ^22^ that contouring uncertainty/variability has a higher impact on DSC for small/thin structures than on large structures. Additionally, image and identification of structure boundaries for OARs can impact contouring accuracy. A previous study^23^ also showed that the average DSC scores achieved by a group of expert physicians for the brainstem is only 0.659; however, the average DSC for the brain was 0.983. Therefore, it can be difficult to assess the quality of a segmentation based on DSC scores alone.

To quantify the relationship between geometric indices and dose, we calculated the Pearson correlation coefficient. All MMs had a Pearson correlation coefficient |r|> 0.50 indicating no significant correlation between geometric indices and dose. Furthermore, these results show that geometric similarity is not an accurate predictor of MM dosimetric variation for HNC radiotherapy. Therefore, it is important to evaluate the dosimetric indices separately for each MM. Figure 3 shows good dosimetric correlation. Yet, contour deviations of several millimeters can impact organ dose in regions with a steep dose gradient — particularly for MMs adjacent to the tumor. Therefore, attention should be given in these regions when checking the integrity of auto‐segmented contours.

Clinically, auto‐segmentation of all masticatory muscles requires 1–2 min using the atlas‐based auto‐segmentation. Furthermore, once a reliable atlas has been generated, this can be shared between centers for reproducible, standardized MM segmentation. Because of this, our auto‐segmentation approach is primed for wide clinical adoption, and has multiple implications for improved patient outcomes, efficiency of care, and standardization of patient contours in the cooperative group setting. While physicians do not routinely provide RT planning constraints or use MMs as avoidance structures, mounting evidences have shown that the dose to the muscles of mastication correlates with trismus and has an impact on patient quality of life.^2^, ^24^ Moving forward, there exists an unmet need to routinely delineate, constrain, and evaluate dose to these masticatory muscles.

Despite the favorable results, the study has several limitations. This is a retrospective analysis of a small patient cohort from a single institution. The gold standard contours used to create the atlas were generated by several expert HNC radiation oncologists. While this approach does improve reproducibility, it is possible that other physicians may delineate the MM structures differently. Future work could incorporate consensus MM segmentations from multiple experts for atlas creation and improved atlas robustness. Furthermore, our current study is experience based on one single institution. Multicenter testing should be implemented for validating the atlas with external patient imaging datasets.

## 5 Conclusion

This study is the first to examine the accuracy of atlas‐based auto‐segmentation for MM delineation, using both multiple geometric and dosimetric indices. We found that atlas‐based auto‐segmentation for muscles of mastication results in geometrically precise automatic organ segmentation and similar organ dose outcomes as compared to manual segmentation. Future work will validate our results on a larger prospective dataset and compare results with other automatic contour generation strategies.

## Conflict of interest

The authors have no relevant conflicts of interest to disclose.
